# Exploring consumer and pharmacist views on the professional role of the pharmacist with respect to natural health products: a study of focus groups

**DOI:** 10.1186/1472-6882-8-40

**Published:** 2008-07-14

**Authors:** Della Kwan, Heather S Boon, Kristine Hirschkorn, Sandy Welsh, Tannis Jurgens, Lynda Eccott, Shirley Heschuk, Glenn G Griener, Jillian C Cohen-Kohler

**Affiliations:** 1 Department of Health Policy, Management and Evaluation, Faculty of Medicine, University of Toronto, Toronto, Canada; 2 Leslie Dan Faculty of Pharmacy, University of Toronto, Toronto, Canada; 3 Department of Sociology, Faculty of Arts and Science, University of Toronto, Toronto, Canada; 4 College of Pharmacy, Dalhousie University, Halifax, Canada; 5 Faculty of Pharmaceutical Sciences, University of British Columbia, Vancouver, Canada; 6 Faculty of Pharmacy and Pharmaceutical Sciences, University of Alberta, Edmonton, Canada; 7 School of Public Health & Department of Philosophy, University of Alberta, Edmonton, Canada

## Abstract

**Background:**

Natural health products (NHPs) such as herbs, vitamins and homeopathic medicines, are currently available for sale in most Canadian pharmacies. However, most pharmacists report that they have limited knowledge about these products which have been regulated in Canada as a specific sub-category of drugs. In this paper, consumers' and practicing pharmacists' perceptions of pharmacists' professional responsibilities with respect to NHPs are examined.

**Methods:**

A total of 16 focus groups were conducted with consumers (n = 50) and pharmacists (n = 47) from four different cities across Canada (Vancouver, Edmonton, Toronto, and Halifax).

**Results:**

In this paper, we illustrate the ways in which pharmacists' professional responsibilities are impacted by changing consumer needs. Many consumers in the study utilized a wide range of information resources that may or may not have included pharmacists. Nevertheless, the majority of consumers and pharmacists agreed that pharmacists should be knowledgeable about NHPs and felt that pharmacists should be able to manage drug-NHPs interactions as well as identify and evaluate the variety of information available to help consumers make informed decisions.

**Conclusion:**

This paper demonstrates that consumers' expectations and behaviour significantly impact pharmacists' perceptions of their professional responsibilities with respect to NHPs.

## Background

Natural health products such as herbs, vitamins and homeopathic products are a growing Canadian product category worth over $400 million annually [1] and are widely available in Canadian pharmacies. Since pharmacists are readily accessible to consumers at the point where they are making decisions about purchasing NHPs, pharmacists are potentially in a good position to provide consumers with evidence-based information about NHPs, especially regarding potential interactions with conventional medications[2]. Pharmacists have the knowledge and experience to help consumers determine when self-medication is appropriate and when the expertise of another health care provider is needed[2]. However, it is not clear that consumers want this kind of advice. With greater access to health-related information, consumers have become more literate, better educated, and increasingly capable of making their own decisions regarding their health care [3-5]. The current situation is the focus of our paper: with NHPs widely available and with engaged and informed consumers demanding access to them, what is the role of pharmacists regarding NHPs? In this paper, we seek to understand how pharmacists' professional responsibilities with respect to NHPs are influenced by consumers who have access to increasing amounts of health information.

### Natural health products in pharmacy practice

Self-medication with natural health products (NHPs), such as herbal medicines and other supplements, has become very popular among Canadians. Based on data from the 2005 Baseline Natural Health Products Survey conducted by Health Canada, seven in ten Canadians have used a NHP at some time in their lives. A majority agree that NHPs can be used to maintain or promote health or to treat illness (68%) [6]. Fewer agree that NHPs are better than conventional medicines (43%) [6]. NHPs are used to treat an existing health condition or in an attempt to prevent illness and often consumed in tandem with conventional medicines [7].

In Canada, NHPs have been governed by the Natural Health Products Regulations since January 2004. Under the Regulations, a NHP is defined as a product found in nature that is "manufactured, sold or represented for use in: (a) the diagnosis, treatment, mitigation or prevention of a disease, disorder or abnormal physical state or its symptoms in humans; (b) restoring or correcting organic functions in humans; or (c) modifying organic functions in humans, such as modifying those functions in a manner that maintains or promotes health" [8] (p. 1573). Products that fall within this category include herbal remedies, homeopathic medicines, vitamins, minerals, traditional medicines (e.g., traditional Chinese medicines), probiotics, amino acids, and essential fatty acids. Tobacco, marijuana and biologics (e.g., blood-based products, insulin) are excluded. By definition, to be considered a NHP, a product must be safe for sale over-the-counter (OTC) and thus be available for self-care and self-selection [8]. More importantly, NHPs are classified as "drugs" at the level of the Federal Food and Drugs Act, which would appear to make them part of the pharmacist's professional scope of practice[8].

Much of the research on NHPs and pharmacy practice to date has focused on describing pharmacists' attitudes towards, and personal use of, NHPs. A systematic review found that Canadian and U.S. pharmacists do not perceive their knowledge of NHPs to be adequate and that a majority of pharmacists would like to receive additional training on NHPs, especially in the areas of interactions, side effects/adverse events, patient counseling, therapeutic uses, and dosing [9]. This is largely the result of the limited NHP-related curriculum in many pharmacy schools until very recently[10]. In addition, survey data reviewed indicate that pharmacists do not routinely document, monitor, or inquire about patients' use of NHPs despite receiving questions about NHPs from patients and other health care providers [9]. Although these surveys provide some description of how pharmacists behave with respect to NHPs, they do not provide any insight into what pharmacists should do or what factors influence how pharmacists' professional responsibilities are determined.

### The patient as consumer

The perspective of the consumer is important to consider when exploring pharmacists' responsibilities in relation to NHPs. An analysis of patients' perceived needs regarding information about NHPs brings into focus the inadequacy of the traditional paternalistic view of the 'patient' as occupying a subject position and demonstrating dependency and unquestioning compliance with medical expertise [11]. Instead, we argue that the majority of consumers interested in NHPs are best understood through the concept of the 'new consumer'. For several decades, the social science and marketing literature have described the existence and influence of "new consumers"[5,12].

New consumers are defined as being information strong, information seeking, and increasingly demanding [4,5]. Compared to 20–30 years ago, current consumers are more literate, better educated, and have more information resources at their disposal [5]. For example, the majority of Canadians are heavy Internet users (56% report being online seven or more hours per week) with researching medical information being one of their most popular Internet activities [13]. Linked to this is the established trend where expert knowledge in areas such as medicine and science is no longer simply accepted on face value. Expert knowledge is now open to skepticism and to challenge on the part of lay people due to an increasing public awareness of the uncertainties that arise from applying group results from clinical trials to individual patients and increasing range of medical care options [14,15].

Much emphasis has been placed in recent literature on the impact of consumerism on the roles and status of healthcare professions in society [15-21]. Eysenbach and Jadad, in their discussion of consumers' increasing access to Internet based resources and the consequences for patient choice, propose a changing role for health professionals [17]. Specifically, they profile a shift from an information 'filter' role of the professional to a 'consumer as partner' model of practitioner-patient relations. In other words, the practitioner and the patient engage in a model of decision making that is more equitable in terms of power relations. Similarly, Fournier argues that the diffusion of professional knowledge to the consumer has eroded the boundary between professionals and clients/lay persons [18]. However, she concludes that the boundary is not eliminated but rather, it is shifted with professions having the capacity to redefine their boundaries as they adapt to changes. Several empirical studies that explored the impact of consumerism on the patient-provider relationship found the situation to be very complex, in that a continual tension exists between seeking dependency and wanting autonomy, which constrains the patient-provider relationship from moving too far in the direction of consumerism [15,19-21]. Although previous literature has provided valuable insights into the impact of consumerism on the roles and status of healthcare professions, only a few studies have investigated how consumer behaviors might be relevant to pharmacy practice [3,5,11].

Most notably, Traulsen and Noerreslet found an increase in 'new consumers' in Danish community pharmacies [5]. The new consumers of medicines actively seek information on side effects, the efficacy and price of products; and in general no longer blindly accept the authority of the pharmacy staff. Seen in light of the theory of risk, the authors concluded that new consumers' behavior is an attempt to minimize the risk of pharmaceuticals, which they have learned about from the media and Internet sources [5]. Hibbert, Bissell, and Ward considered how the presence of new consumers has affected the professional role and status of the community pharmacist in relation to the sale of over-the-counter medicines[11]. They found that consumerism represents a significant challenge to medicine surveillance and professional work in the community pharmacy [11].

Here we investigate how consumers shape pharmacists' responsibilities with respect to NHPs. In the analysis to follow, we use data from focus groups of pharmacists and consumers to explore consumers' and pharmacists' perceptions of the professional responsibilities of the pharmacist with respect to NHPs. Doing so allows us to compare the views of the two groups and to illustrate the ways in which pharmacists' professional responsibilities are influenced by changing consumer patterns.

## Methods

Two focus group discussions with consumers and two with practicing pharmacists were held in University meeting rooms in four different cities across Canada (Vancouver, Edmonton, Toronto, and Halifax) for a total of 16 focus groups. The research was conducted from May to November in 2006. Focus groups were chosen because they provide a forum for participants to discuss a wider range of ideas and issues than would arise in individual interviews [22]. They have also been shown to be an extremely useful tool, especially where researchers seek to access community and public views [23].

A recruitment agency was hired to recruit consumers for the focus groups using random digit dialing. Consumers with a range of age, education, and income were selected. Community pharmacies in Vancouver, Edmonton, Toronto, and Halifax were selected purposefully from the telephone book/Internet listings to recruit a mix of pharmacists practicing in a range of locations, including independent pharmacies, chain drug stores and hospital pharmacies across each city. A pharmacist-investigator contacted each pharmacy by telephone to recruit community pharmacists who met eligibility criteria. (Due to specific ethics requirement in Vancouver, each community pharmacy was faxed a study information sheet before the contact phone call.) In order to recruit both full-time and part-time pharmacists, pharmacies were contacted at different times of the day and at different days of the week. Pharmacists were asked about their years of practice in order to obtain a group with different levels of experience. Hospital pharmacists were recruited by first e-mailing an information letter to the director of selected hospital pharmacies to request recommendations of pharmacists to contact. Each hospital pharmacist was then contacted individually by e-mail.

In total, 50 consumers and 47 pharmacists participated in the study. Please refer to Tables 1 and 2 for a summary of participants' demographics. All the focus groups were led by the same moderator whose main function was to keep the discussion on track, to encourage an open and relaxed discussion, and to probe into areas that needed clarification. The moderator was a pharmacy professor with no ties to community or hospital pharmacy. Current and potential professional (including legal and ethical) responsibilities of pharmacists with respect to NHPs were discussed in each group. See Appendix for specific questions.

**Table 1:** Summary of Pharmacists' Demographics

	**Total Number of Participants**	**Number of Full Time**	**Number of Part Time**	**Number of Pharmacists who have practiced > or = 5 years**	**Number of Pharmacists who have practiced < 5 years**	**Number of Hospital Pharmacists**	**Number of Chain Pharmacists**	**Number of Pharmacists who work for Independent Pharmacies**
**Toronto**	16	13 NB. 1 works both full & part time	2	13	3	4 *1 works in drug information	9 N.B. 1 works in hospital & chain	2
**Halifax**	13	13	1	11	2	0	10	3
**Edmonton**	13	9	3 N.B. 1 just sold pharmacy so was not working at time of data collection	9	4	2	5	5 N.B. 1 just sold pharmacy so was not working at time of data collection
**Vancouver**	5	5	0	4	1	0	2	3

**Table 2:** Summary of Consumers' Demographics

	**Total Number of Participants**	**Age**			**Gender**		**Education**			**Income**	
		
		**25 to 34 NB. Toronto 18 to 34**	**35 to 54**	**Over 54**	**Male**	**Female**	**Completed High School**	**University or College**	**Post Graduate**	**Under 50 000**	**Over 50 000**
**Toronto**	12	9	2	1	7	5	4	6	2	5	7
**Halifax**	12	3	5	4	7	5	3	3	6	5	7
**Edmonton**	14	3	5	6	6	8	4	10	0	6	8
**Vancouver**	12	2	5	5	6	6	1	11	0	2	10

**Figure 1:**
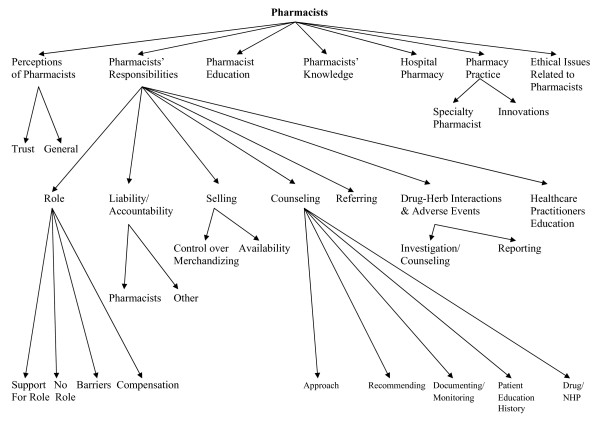
Pharmacist coding tree

The focus group discussions were recorded and transcribed verbatim. Qualitative content analysis was used to identify specific responsibilities for pharmacists with respect to NHPs. Qualitative content analysis involved analyzing the focus group transcripts by categorizing segments of the transcripts into topic areas called "themes" [24]. Each theme was then placed in a topic category based on its content. Large categories were further divided into sub-categories creating a tree-diagram. (See Figure 1 for the pharmacist coding tree.) Each transcript was coded independently by at least two members of the research team who met repeatedly to compare and discuss the coding until consensus was achieved. Computer software (NVivo 7, QSR International Pty. Ltd. ^© ^1999–2006) was used to facilitate this process.

The identity of the participants has been kept confidential by the research team and participants of the groups were instructed not to disclose the identity of other participants and not to discuss anything that occurred during the focus group. All data related to the project has been stored in password projected files and will be destroyed after five years. This research was approved by the Health Sciences Research Ethics Board at the University of Toronto. In addition, ethical approval was provided by each of the Universities where data were collected: Dalhousie University, University of Alberta and University of British Columbia.

## Results

A consistent pattern of responses emerged despite the geographical differences of the participants. Consumers and pharmacists agreed that there is a need for pharmacists to take on a consultative responsibility for NHPs, which takes into account the characteristics of the 'new consumer'. In particular, both agreed that this responsibility is especially important in terms of pharmacists' traditional responsibilities for ensuring patient safety from adverse events and drug interactions associated with NHPs. Not all consumers are information strong though, as we found some evidence for the continued existence of passive patients who rely on their pharmacists for information about NHPs.

### Consumers and NHPs

Consumers in this study generally did not rely on pharmacists for information about NHPs. Many consumers displayed characteristics of the 'new consumer'. They perceived themselves as being capable of making their own decisions regarding the use of NHPs and utilized a wide range of information resources (that may or may not include pharmacists) to make those decisions.

The majority of consumers in our study believed that they were well informed prior to coming to the pharmacy. Many found information from the Internet, friends or family, newspapers, magazines, books, health food stores, or other healthcare professionals (e.g., physicians, naturopaths):

*Well, I'll do research, I'll have a look on the Internet, and then I'll ask people questions. I'll go into their shops, and I'll ask customer service or whoever is working in that area about questions.... Then I'll think about is it going to be good for me, looking at my whole, like what's happening with my own symptoms or whatever, and then I'll choose whether to try it, or whether to not try it*. – Toronto consumer

Some consumers said they never even thought of asking the pharmacist about NHPs:

*It never really would have occurred to me to have asked a pharmacist about anything that was natural. I would have gone to [health food store name]... for that type of information. Most of the stuff I would want to know would probably be more from a nutritional, preventative point of view and that I would think of more of a dietitian or something as a logical source to go for that type of thing as opposed to a pharmacist*. – Vancouver consumer

Pharmacists made the observation that consumers today are information strong and critical of professional advice:

*Well they come with pre-conceived ideas of what is or is not good and... I find myself in the position where I am either explaining why not or trying to understand the basis of the claims that these people are saying these particular products had made about them; I mean it's just very difficult*. – Halifax pharmacist

Consumers also emphasized their skepticism about relying on expert advice:

*We need to be open minded. You have to take some active control yourself, and these are people that you are consulting, not Gods that know everything*. – Edmonton consumer

### The Pharmacist as an NHP "consultant"

Despite the fact that most consumers do not rely on pharmacists for NHP-related advice, the majority of both consumers and pharmacists agreed that pharmacists need to be knowledgeable about NHPs. Our participants suggested that pharmacists could adopt a consultative role to help consumers identify and assess the range of information available, but not necessarily make the final decision for them regarding use.

There was high agreement among consumers that pharmacists need to be knowledgeable about NHPs because usage of NHPs is common among the general public:

*Pharmacists should know that alternative medicines are part of our life, they are reality, and when they sell or fill a prescription, they should be aware that people are likely doing other things as well, and they need to know about it. They need to have a basic understanding about what alternative medicines are*. – Edmonton consumer

More importantly, both consumers and pharmacists agreed that if NHPs are sold in the pharmacy, pharmacists should be responsible for, and knowledgeable about, them.

*I would think that pharmacists should be responsible for the products that their store is selling and not just being one where you need a prescription; they should also be responsible for the vitamins and they should be responsible for the non-prescription drugs... the range of their responsibility is not just the things that are on prescription... *– Halifax consumer

*In my practice, I don't carry things that I don't know about*.

- Edmonton pharmacist

Several consumers argued that pharmacists who are not knowledgeable about NHPs are not fulfilling their professional responsibilities:

*When I talk to pharmacists who don't know about herbal remedies, I kind of think that they must not take their job very seriously...how can you help people if you don't know? *– Vancouver consumer

Our participants suggested that pharmacists could adopt a consultative role to help consumers integrate all types of information when making decisions about the use of NHPs. As one pharmacist described the role:

*I see that one of the roles we have as pharmacists that is very important is to help people to tie in all of their sources of information into something meaningful and useful for themselves. So, we really do need to have some kind of a general education regarding all the different modalities that are available to people so that we can help them make sense of all of the information they are being bombarded with for their own safety, but also for assisting and directing them to alternative choices that might be appropriate for them to use and to still keep it in a safe place and refer them on*. – Edmonton pharmacist

Consumers expected pharmacists to assist them in the appropriate use of NHPs as they do for over-the-counter medications. They would like pharmacists to provide helpful information, but not necessary make the final decision about use:

*I do expect them to give me suggestions. I will read the label but it doesn't always tell me what it's supposed to do for me. So if I expect that for the over the counter stuff, if the herbal things come in, I do expect that they should be able to tell me about the herbal things. If they are going to sell it and I am going to ingest it, I want to know*. – Halifax consumer

Consumers perceived this to be especially important since NHPs can be pharmacologically active and the dosing instructions and safety information for NHPs are often confusing or missing from labels of products.

*...particularly those that are [over] the counter I would think like knowing the most up-to-date literature and why a person would take vitamin C supplement and how much and what are the effects of taking too much...I don't think that information is readily available at the stores...so that may be a good role for pharmacist – to answer some of those questions *– Halifax consumer

In addition, consumers would like pharmacists to help them identify trustworthy information sources.

*Basically he is going to the same source as I could go to, but I would feel a little more comfortable that the pharmacist might be able to differentiate the fake web site from the real web site*. – Edmonton consumer

Pharmacists also described themselves in a consultative role:

*I think we have a responsibility to look up the information for them because we are supposed to be the accessible information provider. We know where to look for it and give them unbiased information. I think we are pretty good at that*. – Vancouver pharmacist

*The idea is to try to give them a bit of education. If they want to take something certainly they don't have to come to a pharmacy.... So I try to say look this is what we know, and I don't want to make the decision for them because it's not my decision to make*. – Vancouver pharmacist

Overall, the majority of consumers and pharmacists agreed that pharmacists should be knowledgeable about NHPs and could adopt a consultative or advisory role to help consumers identify and assess the range of information available about a particular NHP.

### Ensuring patient safety

Although pharmacists recognized the need to respect consumers' expertise and knowledge regarding NHPs, they also placed a great emphasis on their responsibility as pharmacists to ensure patient safety. When asked about their first priority with respect to patient care, the majority of pharmacists clearly identified patient safety, especially with respect to potential drug-herb interactions. This was also identified as a topic that generated many patient questions:

*My first priority is making sure that whatever they are using is not interacting or we are watching for side effects, it is not going to affect their sugars; it is not going to cause any type of unfortunate effect... my first priority as a pharmacist is their safety*. – Edmonton pharmacist

Consumers also agreed that pharmacists are in the best position to manage potential drug-NHPs interactions and to ensure the safe use of NHPs because they have expertise in conventional medications:

*I know the pharmacy here, the pharmacists they have sitting rooms, a little room, and if they give you a prescription that you haven't had before they bring you in and sit you down and discuss it with you, and he's got, you know, records of what I take. If I want to try something new now because I am taking all these other medications, I check with him and he's got it all on the computer and he checks it all out and makes sure that I'm not on anything that is going to make my head burn off or something*. – Edmonton consumer

*He [the pharmacist] knows what I am taking, and because he has that knowledge, I'll ask, I would like to take this, what do you think? Is it going to react with something I am already taking, or you know is it worth it, or do you know of any studies? Because sometimes they will know studies of some new herbal stuff that is being really pushed on the market, and he can tell me it's not worth it*. – Edmonton consumer

Pharmacists were comfortable with letting the consumer make the final decisions only if they were assured that the product would not cause harm:

*So I guess it depends on what they are using it for and whether they have any medical conditions and whether they are on any medications; if they were otherwise healthy and want to try it I would say it's up to you. If they have some major medical conditions and I am not certain if it will cause any adverse reactions then I would probably recommend that they don't try it*. – Toronto pharmacist

*There is always that proviso if you are interested, it is certainly at this point for you, it does not look like it is going to conflict with anything else, if you want to try it, and assess it.... You know I don't know of anything that is going to hurt, so you know let's see how it goes*. – Edmonton pharmacist

When dispensing prescriptions, some pharmacists are proactive and ask for information about NHP use. This allows the pharmacist to check for possible interactions so that they can intervene to protect the consumer. Pharmacists are very unlikely to actively recommend an NHP if a consumer does not first express interest in taking it:

*I ask the patient what kind of herbal product they are on and then I would check the drug interaction, whether they agree with the prescription medication. This I would do, but as far as recommendation, I wait for the customer to ask me, rather then recommend an herb to them*. – Vancouver pharmacist

However, the amount of input a pharmacist can have is ultimately determined by the patient because NHPs are available for self-selection. Thus, consumers may purchase these products without seeking the advice of a pharmacist. More importantly, NHPs are available for sale in a range of retail locations (i.e. health food stores, grocery stores). Consequently, checking for potential drug-NHP interactions is difficult or all but precluded, if the consumer does not approach the pharmacist.

*The problem generally though is people shop at different places. So you know I buy this in here and this in there and I am not telling people when I am buying it; unless somebody points it out to me, I don't know that there is an interaction*. – Toronto consumer

*I think people just grab them and go*. – Edmonton pharmacist

Overall, pharmacists tended to place a great emphasis on ensuring patient safety in terms of their responsibility. In contrast, most consumers emphasized the importance of making their own decisions, while acknowledging that pharmacists could play an important role in helping them to make choices that would not result in harm.

### Consumers as patients

Although many of our participants fit the descriptions of the "new consumer" in the literature, some clearly wanted more of a partnership model of relationship with their pharmacists. What appears to differentiate these consumers is their view of a longer-term relationship between 'patients' and pharmacists:

*[Jay] said the relationship between the pharmacist and the "client" and [Jen] has said the relationship between the pharmacist and "customer"; and to me, I don't want to be a "client". I mean, I know that I am, but I don't want my pharmacist to see me as a "client" or a "customer". I want them to see me more like a "patient" as opposed to a revenue source*. – Toronto consumer

*I do use the internet as well and I have some natural kind of books at home... but I do go to my pharmacist quite a bit...I have been going to the same pharmacist for the last five years since I live near her... so I kind of have a good relationship with her and I trust her and she knows that I have some allergies and things like that so I do talk to her about alternatives sometimes... *– Vancouver consumer

Pharmacists made similar observations:

*I also think it depends on the relationship each pharmacist has with their patient [s]. You know you were talking about how people are just going to go out and take whatever they want anyway. You know I don't find my patients are as much like that. If they trust you and you have a continuity of care relationship, they are more likely to ask you first. A lot of my patients won't take anything, even if they get a prescription from their doctor, they are like, and "Do you think this is okay? You know I don't trust them as much as I trust you". So, it really depends on the kind of practice you have*. – Edmonton pharmacist

In the context of NHPs, the relationship between a consumer and the pharmacist matters. The degree of involvement a pharmacist has in the NHP decision-making process is ultimately controlled by the consumer. But since NHPs are sold in pharmacies, both consumers and pharmacists agreed that pharmacists have a responsibility to provide basic advice about NHPs, especially regarding their safety.

## Discussion

The findings presented in this paper demonstrate that both consumers' and pharmacists' perceptions of the professional responsibilities of the pharmacist with respect to NHPs are affected by the changing behaviour of consumers. Many consumers in the focus groups perceived themselves as being capable of making their own decisions regarding the use of NHPs and utilized a wide range of information resources that may or may not include pharmacists. However, some consumers preferred to adopt a more traditional patient role, seeking a partnership with a particular pharmacist that has earned the consumer's trust.

Both consumers and pharmacists in the study suggested that pharmacists could adopt a consultative role to help consumers integrate different sources of information, but not necessarily make the decision for them about the use of NHPs. The consultative role appears similar to the interpretive or informed choice decision making model [17,25]. In this model, the professional supplies the consumer with relevant information and helps to elucidate and articulate the consumer's values, but does not participate directly in decision making [17,25]. The deliberative or shared decision making model, which researchers and pharmacy leaders advocate to be the best, describes consumers and professionals as active participants in the decision making process with two-way exchange of information and working as partners or friends [17,25,26]. The key difference between the interpretive and deliberative models is that in the interpretive model, the professional does not participate in decision making but in the deliberative model, s/he does. The propensity towards the interpretive, as opposed to the deliberative model, can be understood in the context that NHPs are primarily intended for self-medication by consumers and so it is ultimately the consumers' decision whether or not they choose to use them. One could also argue that in reality, it is the consumer that makes the final decision regarding use of all treatments (including over-the counter products and prescription drug therapy). The data we describe suggest that this is explicitly acknowledged when consumers and pharmacists are discussing NHP use.

Pharmacists in the study tended to place an emphasis on ensuring patient safety, especially with respect to potential drug-NHP interactions, as their first priority in patient care. Most said they would wait for the consumer to take the initiative to ask them for a recommendation about NHPs. Since pharmacists have traditionally taken on the role of gatekeepers in protecting the public from dangerous medicines [27], the 'safety role' can be conceptualized as an extension of this traditional role. In the original paternalistic description of patient-professional interactions, the professional ensures that patients receive the interventions that best promote their health and well-being and adopts the role of main supplier of knowledge [17,25]. What is different in our case is that although most pharmacists were concerned with making sure the products are safe for consumers, they do not perceive themselves to be the main purveyors of information on NHPs. In this context, it is important that pharmacists ask consumers about their NHP use when dispensing prescription medicine so pharmacists can check for interactions.

The 'new consumer' is not a ubiquitous actor, but rather one that emerges more strongly in some contexts than others. For example, the consultative role of pharmacists reflects the impact of the new consumer but where safety considerations emerge, a more traditional paternalistic role of the pharmacist was supported by both consumers and pharmacists in the focus groups. This highlights the tension between seeking dependency and wanting autonomy that exists in the "new consumer" literature [15,20,21]. Even the most information-strong consumer may not have access to detailed information about specific NHP-drug interactions. A minimum responsibility for pharmacists appears to be providing information about NHPs that may interact with prescription drugs. In addition, the consultative role of the pharmacist may come into play as consumers try to sort through large volumes of often conflicting information (and sometimes mis-information) available from a multitude of sources including the Internet.

Like all studies, this one has its limitations. Focus group data is not designed to be generalizable. However, the fact that the themes described in this study were consistent across four geographically disparate Canadian cities suggests that the findings may be applicable to urban areas. It is not clear if Canadians in rural areas, French-speaking Canadians or populations from other countries would express similar opinions.

## Conclusion

In conclusion, when studying the development of professional roles in health care, it is important to consider the consumer perspective and the impact of consumerism on the requirement for health services. Our analysis of consumer and pharmacist focus groups suggests that consumers contribute to shaping the pharmacists' role by using the pharmacist as a consultant and looking to the pharmacist for help with the management of drug-NHPs interactions.

## List of abbreviations

CAM: complementary and alternative medicine; NHP: natural health product; OTC: over-the-counter.

## Competing interests

The authors declare that they have no competing interests.

## Authors' contributions

HSB conceived of the study, obtained funding, participated in data collection, analysis and paper writing. DK participated in data collection, analysis and drafted the initial paper as part of her MSc thesis work. HSB, SW, TJ, and JCC-K participated on DK's MSc supervisory committee and thus provided input on the design of this study. TJ, LE, SH, GGG participated in data collection and paper revisions. KH and SW participated in data analysis and paper revisions. JCC-K participated in paper revisions. All authors read and approved the final manuscript.

## Appendix: Focus group questions

### A. Consumers

#### Views about Current Situation

1. To start with a general question, what are some of your experiences with natural health products (NHPs) like herbal medicines, vitamins and minerals or homeopathic medicines? Give examples.

2. Where do you go for information about NHPs?

• PROBE: What kind of information do you usually ask about?

3. Where do you usually purchase NHPs?

• PROBE: Do you buy NHPs at the pharmacy? Why or why not?

• PROBE: How do you decide where to buy NHPs?

• PROBE: Do you think it is safer to buy NHPs at the pharmacy?

4. Have you ever talked to a pharmacist about NHPs? Why or why not? Give examples.

• PROBE: What do pharmacists currently do well? What do pharmacists currently do poorly?

#### Prescriptive Views (i.e. how they think things 'should' be)

5. What would you like to see pharmacists do with respect to NHPs?

• PROBE: Should pharmacists sell NHPs? Why or why not?

• PROBE: Should NHPs be sold only in the pharmacy?

• PROBE: Should pharmacists recommend NHPs to patients? Under what circumstances?

• PROBE: What kind of information should pharmacists provide to patients about NHPs (e.g., instructions for use, potential adverse effects, whether there is evidence to support the efficacy and safety of the product, etc.)?

• PROBE: Should pharmacists be responsible for detecting interactions between NHPs and drugs?

#### Inter-professional Responsibilities

6. Who do you identify as the (other/potential) professional experts in the area of NHPs?

• PROBE: Are these experts for NHPs in general or only for particular NHPs?

• PROBE: What do you think is the role of the physician/nurse/dietitian/naturopath with respect to NHPs?

#### In Closing

7. Do you have any advice to give to the profession of pharmacy in regards to what their professional responsibilities for NHPs should be?

### B. Practicing Pharmacists

#### Views about Current Situation

1. To start with a general question, what are some of your experiences with natural health products (NHPs) like herbal medicines, vitamins and minerals or homeopathic medicines? Give examples.

2. What do you currently do with respect to NHPs?

• PROBE: Do you ask your patients if they use NHPs? Why or why not?

• PROBE: Do you recommend NHPs to patients? Why or why not?

• PROBE: Do you provide counseling on NHPs? Why or why not and what information do you provide?

• PROBE: Do you check for drug-herb interactions? Why or why not and what would you do if you suspect one?

3. What training or education have you had about NHPs?

#### Prescriptive Views (i.e. how they think things 'should' be)

4. What general legal and ethical responsibilities (if any) do you think pharmacists should have with respect to natural health products?

• PROBE: Should pharmacists sell NHPs? Why or why not? What legal and ethical responsibilities do pharmacists have if they sell NHPs?

• PROBE: Should NHPs be sold only in the pharmacy? Why or why not?

• PROBE: Should pharmacists be liable for the safety and quality of the NHPs that are sold at the pharmacy?

• PROBE: Are there particular kinds of NHPs that pharmacists should be responsible for?

• PROBE: Are NHPs a part of providing pharmaceutical care? Why or why not?

• PROBE: What kind of information do you think should be provided in counseling about NHPs (e.g., instructions for use, potential adverse effects, whether there is evidence to support the efficacy and safety of the product, etc.)?

• PROBE: Should pharmacists recommend NHPs to patients? Under what circumstances?

• PROBE: Should pharmacists be responsible for detecting interactions between NHPs and drugs? Why or why not?

• PROBE: Should pharmacists provide information about NHPs to other members of the health care team (i.e., physicians, nurses, etc.)? Why or why not?

• PROBE: Should all pharmacists have the same responsibilities or are there potentially different responsibilities for different types of pharmacists?

5. What training do you think is required for pharmacists on NHPs?

• PROBE: In your opinion, what is the best way to help pharmacists gain the knowledge and skills necessary to perform the responsibilities discussed?

6. Do you think NHPs fall within the pharmacist's scope of practice as it is currently defined? Why or why not?

7. What policy changes do you think are needed in order for pharmacists to adopt the responsibilities discussed (i.e., revise/expand practice standards, code of ethics, and scope of practice)?

8. What challenges do you anticipate would be encountered by pharmacists in adopting the responsibilities discussed?

#### Inter-professional Responsibilities

9. Who do you identify as the (other/potential) professional experts in the area of NHPs?

• PROBE: Are these experts for NHPs in general or only for particular NHPs?

• PROBE: What do you think is the role of the physician/nurse/dietitian/naturopath with respect to NHPs?

#### Product Regulation

10. What have you heard about the new natural health product regulations? Do you think these will have any impact on the pharmacist's professional role with respect to NHPs? Why or why not?

#### In Closing

Do you have any advice to give to the profession of pharmacy in regards to what their professional responsibilities for NHPs should be?

## Pre-publication history

The pre-publication history for this paper can be accessed here:


